# Physical Education-Based Stretching During Warm-Up, Cool-Down, or Both on Back-Saver Sit-and-Reach Scores in Schoolchildren

**DOI:** 10.3390/jfmk10040383

**Published:** 2025-10-02

**Authors:** Rafael Merino-Marban, Iván López-Fernandez, Daniel Mayorga-Vega

**Affiliations:** Departamento de Didáctica de las Lenguas, Las Artes y el Deporte, Facultad de Ciencias de la Educación, Universidad de Málaga, 29071 Málaga, Spain; ivanl@uma.es (I.L.-F.); dmayorgavega@uma.es (D.M.-V.)

**Keywords:** flexibility, children, intervention, hamstring extensibility, flexibility field test

## Abstract

**Objectives:** The aim of this study was to compare the effects of eight-week hamstring stretching programs, implemented at different times during physical education classes (i.e., warm-up, cool-down, and both periods), on primary schoolchildren’s back-saver sit-and-reach scores. **Methods:** A total of 275 schoolchildren (141 females and 134 males; age 8.82 ± 1.63 years) were divided into four groups: the WUG performed stretching during warm-up, the CDG during cool-down, and the MXG during both. The NSG followed the standard classes of physical education without any stretching. During physical education classes WUG, CDG, and MXG performed a 4 min stretching program twice a week. Hamstring extensibility was assessed before and after the program using the back-saver sit-and-reach test. **Results:** The CDG is the one that achieved statistically significant improvements compared with the WUG, MXG, and NSG (*p* ≤ 0.01; d = 0.50–0.71). Moreover, the CDG statistically increased the percentage of schoolchildren achieving healthy hamstring extensibility from pre-intervention (49%) to post-intervention (66%). **Conclusions:** This knowledge could guide teachers to design programs that guarantee feasible and effective development of hamstring extensibility in the physical education setting.

## 1. Introduction

Physical education (PE) plays a crucial role in promoting physical fitness, particularly in the development and maintenance of key health-related fitness components such as cardiovascular endurance, strength endurance, and flexibility [1]. In the same vein, Lopes et al. [2] highlighted the importance of promoting and developing flexibility, as well as the other health-related physical fitness components in schoolchildren, to reach adequate levels of motor competence. Flexibility is a quality that, in the absence of intervention, tends to decline, beginning in childhood [3,4]. In this regard, Mula and Sainz de Baranda [5], in their review, emphasize the importance of systematically incorporating stretching exercises into PE curricula to counteract this decline. The risk of musculoskeletal pain and disability due to loss of flexibility during growth is significant for children, particularly in primary school [6]. Prior research shows that stretching exercises throughout an academic year can help prevent the loss of flexibility and a decrease in musculoskeletal disorders, joint pain, injuries, and obesity in schoolchildren, all of which contribute to their locomotor health [3].

Flexibility, defined as the capacity to perform movements through a wide range of motion [7], confers important health benefits in children. Insufficient flexibility may impair motor competence in youth [8], and given the apparent developmental window for flexibility during childhood, systematic stretching interventions are recommended during these formative years [9].

According to various studies, hamstring tightness is a relatively common musculoskeletal condition among schoolchildren [5,10,11,12]. This condition is particularly amenable to prevention and treatment through stretching and postural correction [11]. The school environment is ideal for implementing preventive strategies to address hamstring muscle shortening, as it allows for the optimization of conditions and the provision of sustained feedback, reaching a large proportion of the student population [4]. Additionally, Peña et al. [13] support the implementation of hamstring flexibility intervention programs in PE for schoolchildren to enhance physical performance and prevent potential injuries.

Numerous studies have assessed the effectiveness of hamstring stretching programs implemented during PE lessons for primary schoolchildren [12,14,15,16,17,18,19,20]. Unfortunately, currently only one study has specifically examined the impact of implementing hamstring stretching exercises at different moments during the PE lesson [14]. However, the sample size was small (73 schoolchildren), there was no experimental group (EG) combining stretching during both warm-up and cool-down, and the EGs did not improve hamstring extensibility.

The timing of stretching within a PE lesson is not a trivial methodological choice but a variable with physiological and performance implications. Evidence shows that static stretching before physical activity may transiently reduce neuromuscular performance in youth, affecting explosive strength, jump height, and sprint speed [21,22,23].

In contrast, stretching after activity, when muscles are warm and under less neuromuscular demand, may optimize viscoelastic properties, favoring long-term flexibility gains without impairing performance [21,24]. It may also help restore optimal resting length and counteract adaptive shortening during growth [3,5].

Although some studies applied stretching in both warm-up and cool-down [11,19], few compared timings in school settings. Mayorga-Vega et al. [14] suggested a possible advantage of cool-down stretching, though with methodological limitations. Therefore, directly comparing warm-up, cool-down, and combined strategies while controlling total volume is essential. This study addresses that gap by examining their effects on hamstring extensibility in primary schoolchildren.

Consequently, the aim of this study was to compare the effects of eight-week hamstring stretching programs, implemented at different times during PE classes (i.e., warm-up, cool-down, and both periods), on primary schoolchildren’s back-saver sit-and-reach (BSSR) scores.

## 2. Methodology

For practical reasons and the nature of the present study (i.e., intervention focused on natural groups in a school setting), a cluster-randomized controlled design was used [15]. Balanced by school and grades, natural classes were assigned randomly to form one of the following study groups: warm-up group (WUG), cool-down group (CDG), mixed group (MXG), and non-stretching group (NSG). The randomization was carried out after the familiarization period and the baseline assessment. The study protocol was first approved by the Ethical Committee of the University of Malaga (reference approval number: 105-2022-H), approved on the 9 November 2022. After the school approvals were obtained, and before starting the intervention, children’s verbal informed assents and their legal guardians signed written informed consents were obtained.

### 2.1. Participants

A total of 289 schoolchildren (141 females and 148 males) from four second-, four fourth-, and four sixth-grade classes at two public primary schools were invited to participate in this study. Of these, 275 agreed and met the inclusion criteria, which were (a) absence of orthopedic disorders, including episodes of hamstring and/or lumbar injuries, fractures, surgeries, or pain in the spine or hamstring/lumbar muscles during the previous six months [25]; (b) verbal consent from the schoolchildren; and (c) signed informed written consent from parents or legal guardians. The exclusion criteria were (a) incomplete flexibility evaluations, and (b) attendance below 90% in PE classes during the intervention period.

### 2.2. Measures

All the measures were performed during the regularly scheduled PE classes. Prior to the intervention, information about participants’ gender and age was collected. Then, anthropometric measurements were also taken during the same class. Afterward, the flexibility test was applied before and after the stretching program in order to examine whether possible changes took place.

### 2.3. Anthropometric Measurements

Participants’ body mass and height were measured and then the body mass index (BMI) was calculated as body mass/height squared (kg/m^2^). During the measurement of body mass and height, participants wore shorts and T-shirts and were barefoot. For the body mass measurement, once the scale read zero, the participant stood in the center of the scale without support, distributing body mass evenly on both feet. For the height measurement, the students stood with their feet and heels together, their buttocks and upper part of the back touching the scale, and with their head placed in the Frankfort plane. The average of two measurements for both body mass and height were retained [26]. A Tanita UM-050 digital weighing scale (Tanita UK Ltd., Middlesex, UK) was used to assess body mass. Height was assessed using a stadiometer (SECA Leicester, Birmingham, UK).

### 2.4. Back-Saver Sit-and-Reach Score

Participants’ hamstring extensibility was estimated by the BSSR test. The BSSR was carried out by the same evaluator and instrument (a box Eveque, Eveque Leisure Equipment Ltd., Cheshire, UK). Additionally, the measurements were performed in an indoor sports facility under similar environmental conditions, on the same day of the week and at the same time for each participant. Schoolchildren were also reminded to avoid any exhausting physical activity 48 h prior to each evaluation session.

**Figure 1:**
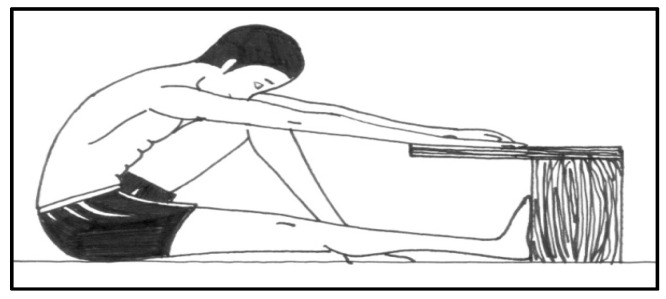
Performance of the back-saver sit-and-reach test.

The application of the BSSR test was always carried out at the beginning of class, during PE hours, when students performed a warm-up of five minutes in each assessment session. The standardized warm-up consisted of three minutes of jogging at low intensity followed by two static and bipodal hamstring stretching exercises (two 20 s sets of each exercise). Then, the participants, in their sportswear and barefoot, were assessed by the BSSR test. The participants sat with one leg (not assessed) flexed at the knee and the other leg subject to the study extended with their foot against the box. From this posture, the child had to bend the trunk forward slowly and progressively (no rebounds) in order to reach the greatest possible distance [27]. Researchers encouraged the participants through verbal reinforcement during the execution of the test. In the maximum flexion posture, the participant had to remain still for at least two seconds and the distance was recorded in centimeters, taking the value to the nearest 0.5 cm (Figure 1). A researcher instructed the participants and held the assessed leg above the knee to avoid a knee flexion. A 15 cm mark occurred when a participant could reach the foot, while positive values were attributed as one exceeded this point, and negative values attributed when the foot was not reached. Two trials were performed 30 s apart, and the average was retained. The score was calculated as the average of both sides.

The BSSR test represents a healthier alternative to the traditional sit-and-reach, since stretching the two hamstrings simultaneously could produce an overstretching of the lumbar region [28]. It has an acceptable validity, shows a high degree of reliability, and has further characteristics such as low cost, easy to administer, and the ability to measure a large number of people [29].

### 2.5. Procedure

A stretching intervention program was applied to WUG, the CDG and the MXG during PE classes. These group participants performed a stretching intervention program twice a week on non-consecutive days for 8 weeks. Teachers alternated stretches in the two weekly sessions [16] so that they did not repeat exercises within the same week. In the PE planning, this kind of intervention implementation is called an “intermittent teaching unit” [30]. The stretching program was conducted and supervised by the same PE teacher for the groups from the same school. The WUG students performed four minutes of stretching at the end of the warm-up, the CDG students performed four minutes at the end of the cool-down, and the MXG students performed two minutes at the end of the warm-up and two minutes at the end of the cool-down. The NSG students followed the same standard PE classes without performing any stretching exercise.

### 2.6. Familiarization Period

PE teachers were responsible for implementing the program. Six weeks before the initial assessment, they were handed a report on the data collection protocol and flexibility program to discuss with the research team. To standardize the implementation of the program, PE participants and teachers participated in training sessions in order to become familiarized with the exercises, the test, and the organization of the program. Such familiarization was completed three weeks prior to the training program in one session. When they did not perform well during the test, the students repeated the stretching exercises and BSSR test twice, and the NSG students only performed the BSSR test twice.

### 2.7. Hamstring Stretching Program

**Figure 2:**
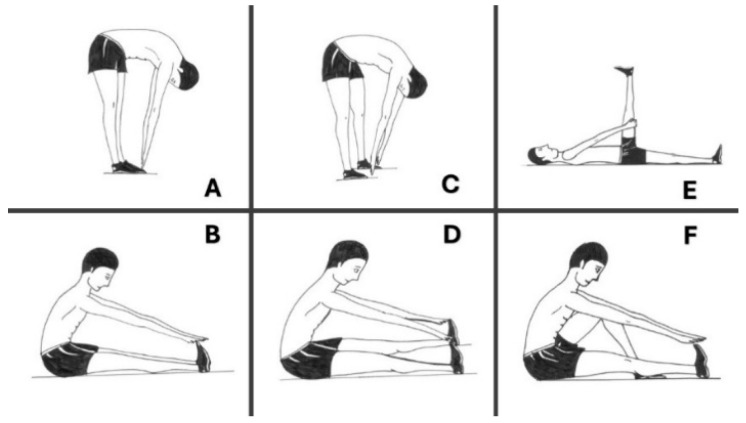
The six stretching exercises performed during the physical education-based hamstring stretching program were (**A**) standing with feet together; (**B**) sitting with feet together; (**C**) standing with feet shoulder-width apart; (**D**) sitting with feet shoulder-width apart; (**E**) lying on back with one leg extended, and (**F**) sitting with only one leg extended. All exercises were supervised by PE teachers to ensure correct execution, especially maintaining spinal alignment with a neutral spine and anterior pelvic tilt, in order to prevent lumbar spine rounding and to guarantee safe performance.

Six different stretching exercises were performed during the intervention program (Figure 2: session 1: exercises A, C, and E; session 2: exercises B, D, and F). Within each session, participants began with unilateral exercises and ended with bilateral exercises. Unilateral exercises entailed three repetitions on each leg and, after that, two bilateral exercises were performed. Thus, there were two minutes of unilateral exercises and two minutes of bilateral exercises in each PE session. Participants completed three repetitions of each exercise, for twenty seconds per repetition and with a five second break between repetitions. A total of 240 s was spent stretching in each session and, if we consider that some exercises were unilateral, each leg received 180 s of stretching. For all the stretching exercises, the children flexed forward at the hip, maintaining the spine in a neutral position until a gentle stretch was felt in the hamstrings. The subjects tilted their pelvis forward to create a lordosis in the lumbar spine [1]. The knee of the stretching leg was fully extended and without hip rotation. The stretching positions were held gently until the end point of the range was reached (i.e., stretch to the point of feeling the tightness of the hamstring muscles, but without pain). Once this position was achieved, the children held it for 20 s (static technique). All stretching exercises were performed under the close supervision of the PE teachers, who provided continuous verbal cues to ensure maintenance of a neutral spine and anterior pelvic tilt. Children were reminded that inadequate posture, particularly rounding of the lumbar spine, could compromise spinal health and reduce the effectiveness of the stretch.

All the participants were urged to maintain their normal levels of physical activity outside of the supervised setting during the intervention period. During the stretching program period all the students participated in their standard PE lessons. However, the NSG followed the standard PE program without performing hamstring stretches. Furthermore, the participants in the NSG were unaware of the purpose of the study.

### 2.8. Data Analysis

Descriptive statistics (means and standard deviation/standard error or percentage) were computed for general characteristics of the participants and the dependent variable. Firstly, all statistical test assumptions were checked by common procedures (e.g., histograms and Q-Q plots for normality) and met. Afterward, the comparison of the effect of the hamstring stretching programs on schoolchildren’s BSSR scores was examined. Since the implementation of the missing data requires strong assumptions that are hard to justify, “complete case” analyses including only those whose outcomes were known were used (i.e., *n* = 237) [31]. Since the unit of randomization and intervention was the class, a Multi-Level Linear Model (MLM) with participants nested within classes as random effects, and with the between-group factor group (WUG, CDG, MXG, and NSG) as fixed effects on the change in BSSR scores (i.e., post-intervention–pre-intervention) was selected (i.e., one-way nested ANOVA) [32]. The maximum likelihood estimation method was used. Subsequently, the post hoc between-group pairwise comparisons with the Bonferroni adjustment were carried out. Since none of the potential confounding variable explored (i.e., gender, grade, body mass, height, body mass index, and pre-intervention BSSR scores) showed statistical significance, covariables were not included. Moreover, the results of the MLM with participants nested within classes as random effects, and with the between-group factor group (WUG, CDG, MXG, NSG) as fixed effects on the pre-intervention BSSR scores, followed by the pairwise comparisons with the Bonferroni adjustment showed that there were no statistically significantly differences between groups (*p* > 0.05).

When analyzed separately by group, the results of the MLM with participants nested within classes as random effects, and with the within-group factor time (pre- and post-intervention) as fixed effects, showed that a statistically significant improvement in BSSR scores was observed only in the CDG (F = 11.030, *p* = 0.02).

Furthermore, separately for each group, the exacted McNemar’s test was used in order to examine the effect of the hamstring stretching programs on the percentage of schoolchildren achieving healthy hamstring extensibility (i.e., ≥13 cm, that means, ≥−2 cm regarding the tangent of the feet) [16,33]. Effect sizes were estimated using the Cohen’s *d* (continuous variables) and Cramer’s V (categorical variables) for the pairwise comparisons. All statistical analyses were performed using SPSS version 25.0 for Windows (IBM^®^ SPSS^®^ Statistics). The statistical significance level was set at *p* < 0.05.

## 3. Results

From the 289 schoolchildren that were invited to participate in the present study, 275 agreed and met the inclusion criteria (WUG = 67; CDG = 62; MXG = 72; NSG = 74). Since 38 of them did not satisfactorily pass the exclusion criterion, in the end, 237 participants were analyzed (WUG = 57; CDG = 55; MXG = 61; NSG = 64). General characteristics of the analyzed participants are shown in Table 1. BSSR’s scores in the pre- and post-intervention of the analyzed participants are shown in Table 2.

**Figure 3:**
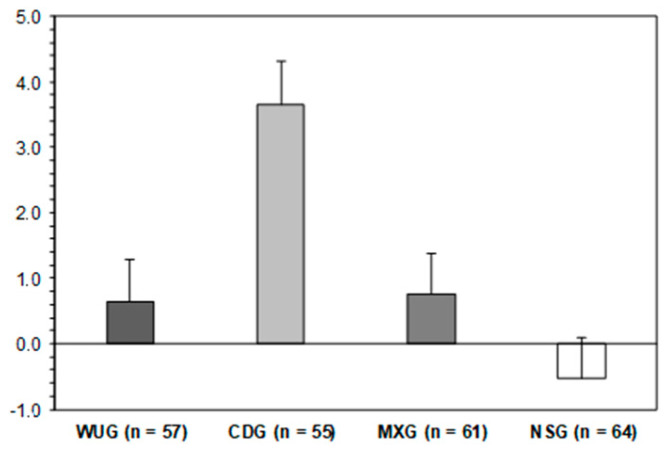
Change pre- and post-intervention in back-saver sit-and-reach. WUG: warm-up group; CDG: cool-down group; MXG: warm-up + cool-down group; NSG: no-stretching group.

As reported in Figure 3, the MLM results showed overall statistically significant effects on schoolchildren’s change in BSSR scores (−2*LL* = 1426.352; *F* = 7.550; *p* < 0.001). Afterwards, the between-subject pairwise comparisons with the Bonferroni adjustment showed that the CDG (*M* = 3.7; *SE* = 0.7) schoolchildren experienced a statistically significant improvement compared with the WUG (*M* = 0.6; *SE* = 0.7; *p* = 0.008; *d* = 0.51), MXG (*M* = 0.8; *SE* = 0.6; *p* = 0.010; *d* = 0.50), and NSG (*M* = −0.5; *SE* = 0.6; *p* < 0.001; *d* = 0.71). However, between the WUG, MXG, and NSG schoolchildren, statistically significant differences were not found (*p* > 0.05; *d* = 0.20 and 0.22, respectively).

**Figure 4:**
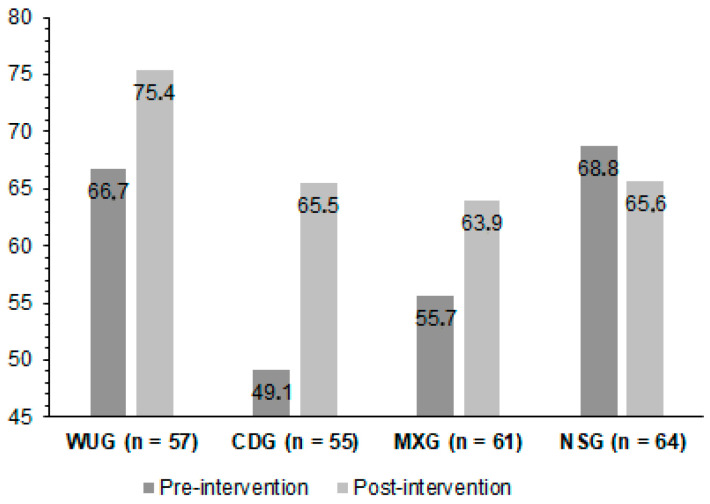
Schoolchildren achieving healthy hamstring extensibility in back-saver sit-and-reach (%). WUG: warm-up group; CDG: cool-down group; MXG: warm-up + cool-down group; NSG: no-stretching group.

As illustrated in Figure 4, the results of the exact McNemar’s test showed the CDG significantly increased the percentage of schoolchildren achieving a healthy hamstring extensibility from pre-intervention to post-intervention (*p* = 0.022, Cramer’s V = 0.560). However, for the WUG, MXG, and NSG schoolchildren, statistically significant differences were not found (*p* > 0.05).

## 4. Discussion

The aim of this study was to compare the effects of eight-week hamstring stretching programs, implemented at different times during PE classes (i.e., warm-up, cool-down, and both periods), on primary schoolchildren’s BSSR scores. The findings of the present study showed that the CDG schoolchildren experienced a statistically significant improvement compared with those from the WUG, MXG, and NSG. However, between WUG, MXG, and NSG schoolchildren, statistically significant differences were not found. Another important outcome is that the CDG increased, statistically, the percentage of schoolchildren achieving healthy hamstring extensibility from pre-intervention (49%) to post-intervention (66%). However, for the WUG, MXG, and NSG schoolchildren, statistically significant differences were not found. These results suggest that performing stretching exercises in the cool-down period is far more effective than doing them in the warm-up or splitting the stretching time in the warm-up and cool-down periods. It could be that performing the stretching exercises in the cool-down period relaxes the muscles worked during PE classes, returning them to their previous length and even improving it, which might not happen when stretching exercises are performed before the main part of the PE lesson. Moreover, if these exercises are particularly hard or intense, they may even increase the shortening of the trained muscles [34].

These results are consistent with other studies involving hamstring stretching programs in primary school PE classes [4,11,12,14,15,16,17,18,19,20,35] with different protocols ranging between 8 to 32 weeks, two to four times per week, holding each stretch for a duration of 15 to 20 s for a total of 180 to 420 s.

If we carefully analyze the studies mentioned in the previous paragraph, we observe that the studies between 8 and 10 weeks had positive results in the program when conducting the stretches during the cool-down phase of the PE lesson [15,16,18,20] except in the study by Mayorga-Vega et al. [14], in which the WUG and CDG did not show post- and pre-intervention differences on hamstring extensibility and the NSG showed statistically significant lower scores than the WUG and CDG, although CDG schoolchildren obtained a slightly higher magnitude effect than the WUG when compared with the NSG (0.67 vs. 0.56). Compared to Mayorga-Vega et al. [14], in the present study, there are certain differences that would justify the different results. The present study used a much larger sample (275 vs. 73 schoolchildren). In addition to the EGs performing stretching either during warm-up or cool-down, there was a third EG that performed stretching in both phases. Additionally, in this case, more emphasis was placed on the technical execution of the exercise, the schoolchildren tilted the pelvis forward to create lordosis in the lumbar spine during the stretching exercises. Also, according to the study by Sullivan et al. [24], performing pelvic tilt anteversion during stretching exercises is a key factor in increasing hamstring extensibility.

Sixteen weeks of stretching during the warm-up are required for the EG to show improvements in stretching [12]. When stretching is conducted throughout the academic year twice a week, divided between the warm-up and the cool-down, 3 min in the warm-up and 2 min in the cool-down, EGs usually improve [4,11,19,35]. However, if the stretches are conducted throughout the academic year, only in the cool-down, even if it is just one session (3 min) per week, the EG shows significant improvement [17].

Regarding the magnitude effects of the intervention, the effect size of the present study was moderate-high for CDG (*d* = 0.70) compared to the NSG and moderate compared to WUG (*d* = 0.50) and MXG (*d* = 0.51), indicating that the stretching program was effective to improve BSSR scores. Similar to the present results, all the previous studies carrying out short-term PE-based hamstring stretching programs (8–10 weeks) obtained, on average, moderate effect sizes (*g* = 0.35 to 0.67) [14,15,16,18,20].

When increasing a training factor such as the duration, the magnitude was higher for the mid-term hamstring stretching programs (16 weeks) (*g* = 0.86, 0.85–0.88) [12], and even higher for those with long-term hamstring stretching programs (whole school year, 31–32 weeks) (*g* = 0.94, 0.85–2.06) [1,4,11,19,35]. In the same way, increasing the frequency of the hamstring stretching program could have positive consequences on the magnitude effects. In regard to the frequency of the hamstring stretching program, Santonja et al. [11] found that when the schoolchildren performed four sessions per week instead of two, the magnitude effect doubled (twice a week, *g* = 0.85; four times a week, *g* = 1.53; both 5 min per session, 31 weeks of duration). Also, Mayorga-Vega et al. [17] found that when schoolchildren performed one session per week, the magnitude effect was smaller (*g* = 0.60; 3 min per session, 32 weeks of duration). In any case, according to Valentine and Cooper [36], we must be aware that in education research, even lower values of effect size (0.30) could be considered of practical relevance.

All primary school hamstring stretching programs reviewed used a static technique, as indicated in our research. This technique is recommended in the school setting because of its ease, safety, and because it allows greater control of the spine alignment [11]. Intensity employed in all studies was mild except in Coledam et al. [12], where participants reached the onset of muscle or joint discomfort.

## 5. Strength and Limitations

The main strength of the present study was that this is the first study that examines the effect of hamstring stretching programs, implemented at different times during PE classes (i.e., warm-up, cool-down, and both periods), on primary schoolchildren’s back-saver sit-and-reach scores. Another strength is the cluster-randomized controlled trial design used as it reduces bias and enhances internal validity. Additionally, the large sample analyzed, 237 participants, improved statistical power and generalizability. Likewise, the participants and teacher training and familiarization with the exercises and the test ensured program consistency. Moreover, because of the nature of the context (i.e., school) and with the objective of keeping the ecological validity, the use of a cluster-randomized controlled trial design (balanced by grade) was more appropriate for the present research objective [18]. Finally, the evaluation of the effect of the hamstring stretching programs with a Multi-level Linear Model, with participants nested within classes and measurements nested within participants as random effects, represents an advancement with respect to the commonly applied analyses [32].

This study also has some limitations that should be acknowledged. Firstly, an eight-week intervention may not reflect long-term outcomes, and the lack of follow-up prevents us from knowing the levels of retention in hamstring extensibility. However, considering the large volume of objectives that have to be developed throughout the academic year with a very limited time for the PE subject [37], the purpose was to perform a real study that would be feasible to perform in the context of PE. Furthermore, the results are specific to primary schoolchildren, so they may not apply to adolescents or other age groups. In addition, there was limited control of covariates, since socioeconomic level, physical activity levels, and motivation were not considered. In addition, although teachers were trained and familiarized to instruct and verbally correct the children to maintain a neutral spine and anterior pelvic tilt during the stretching exercises, we did not carry out an objective verification of postural alignment. This aspect may therefore be considered a limitation of the study. Additionally, although all exercises in the present study were performed under teacher supervision with instructions to maintain a neutral spine and pelvic tilt, it should be acknowledged that poor execution of forward trunk flexion stretches may increase mechanical stress on the lumbar spine and the risk of intervertebral disc strain. Therefore, postural education and close supervision are essential components of school-based stretching programs to ensure both safety and effectiveness [25]. Future studies should include a post-intervention tracking of flexibility retention, and greater control of covariates. Furthermore, carrying out similar interventions in other population groups and using other stretching techniques would contribute to the expanding literature on flexibility programs. Additionally, although the present intervention focused on the physiological effects of stretching, we acknowledge that motivational and engaging pedagogical approaches may be crucial for long-term adherence and meaningful learning in PE. Future programs should consider combining evidence-based stretching protocols with student-centered methodologies to maximize both effectiveness and sustainability.

## 6. Conclusions

An eight-week program of hamstring stretching performed during the cool-down in PE classes achieved improved scores in the back-saver sit-and-reach. The effect size for the group that stretched during the cool-down was 0.71, and for the groups that stretched during the warm-up and in both phases, it was 0.20 and 0.22, respectively, compared with the NSG. The WUG increased from 66.7% to 75.4%, the CDG increased from 49.1% to 65.5%, the MXG increased from 55.7% to 63.9%, and the NSG slightly decreased from 68.8% to 65.6%, but only the CDG increased, statistically, the percentage of schoolchildren achieving a healthy hamstring extensibility from pre-intervention to post-intervention. Hence, cool-down stretching yielded the greatest improvements in hamstring flexibility, supporting its prioritization in PE curricula over warm-up or mixed-phase stretching

## 7. Practical Application

PE teachers should incorporate stretching during the cool-down period into their classes to enhance flexibility. This study indicates that stretching during the cool-down phase is effective compared to stretching during both the warm-up and cool-down and stretching only during the warm-up. As a result, programs focusing on post-exercise flexibility should prioritize cool-down stretches to maximize flexibility improvements.

Additionally, previous studies have shown that the inclusion of static stretching exercises during the warm-up negatively affects the posterior performance in important parameters such as explosive strength in children [22,23]. Hence, for all the above-mentioned reasons, it seems to be more reasonable for PE teachers to improve students’ flexibility during the cool-down period.

School-based PE curricula should be revised to include specific recommendations on stretching. This study supports the inclusion of cool-down stretches to improve flexibility and flexibility maintenance, which can contribute to overall student well-being and performance in sports activities.

For exercise regimen where time is a limiting factor (PE setting), this study supports the idea that cool-down stretching alone can be more effective than combining warm-up and cool-down stretching.

Ultimately, stretching should not be treated as an optional complement but rather as an essential and systematically integrated component of K–12 physical education, given its proven role in safeguarding children’s musculoskeletal health.

## Figures and Tables

**Table 1:** General characteristics of the analyzed participants.

	WUG (*n* = 57)	CDG (*n* = 55)	MXG (*n* = 61)	NSG (*n* = 64)	Total (*N* = 237)
Gender % (females/males)	63.2/36.8	52.7/47.3	37.7/62.3	62.5/37.5	54.0/46.0
Grade % (2nd/4th/6th)	40.4/29.8/29.8	40.0/38.2/21.8	36.1/34.4/29.5	35.9/32.8/31.3	38.0/33.8/28.3
Body mass (kg)	38.2 (10.9)	36.4 (12.0)	36.1 (9.8)	37.9 (10.5)	37.2 (10.8)
Body height (cm)	138.4 (13.3)	136.8 (11.4)	136.9 (10.1)	137.3 (9.8)	137.3 (11.1)
BMI (kg/m^2^)	19.6 (3.4)	19.0 (4.1)	19.0 (3.2)	19.7 (3.8)	19.3 (3.6)

WUG: warm-up group; CDG: cool-down group; MXG: warm-up + cool-down group; NSG: no-stretching group; BMI: body mass index. Continuous data expressed as mean ± standard deviation.

**Table 2:** BSSR’s scores in the pre- and post-intervention of the analyzed participants.

	Pre-Intervention Mean (SD)	Post-Intervention Mean (SD)
Warm-up group (*n* = 57)	16.6 (5.7)	17.2 (6.7)
Cool-down group (*n* = 55)	13.4 (6.0)	17.0 (10.2)
Warm-up + Cool-down group (*n* = 61)	13.9 (4.6)	14.7 (5.9)
No-stretching group (*n* = 64)	16.1 (6.4)	15.6 (6.9)

BSSR: back-saver sit-and-reach. SD: standard deviation.

## Data Availability

The data that support the findings of this study are openly available in [RIUMA] at https://dx.doi.org/10.24310/riuma.38319, reference number [10.24310/riuma.38319].
